# Bioactive Compounds as Inhibitors of Inflammation, Oxidative Stress and Metabolic Dysfunctions via Regulation of Cellular Redox Balance and Histone Acetylation State

**DOI:** 10.3390/foods12050925

**Published:** 2023-02-22

**Authors:** Hyunju Kang, Bohkyung Kim

**Affiliations:** 1 Department of Food and Nutrition, Keimyung University, Daegu 42601, Republic of Korea; 2 Department of Food Science and Nutrition, Pusan National University, Busan 46241, Republic of Korea

**Keywords:** bioactive compounds, anti-inflammation, anti-oxidative stress, anti-metabolic syndrome, cellular redox balance, histone acetylation

## Abstract

Bioactive compounds (BCs) are known to exhibit antioxidant, anti-inflammatory, and anti-cancer properties by regulating the cellular redox balance and histone acetylation state. BCs can control chronic oxidative states caused by dietary stress, i.e., alcohol, high-fat, or high-glycemic diet, and adjust the redox balance to recover physiological conditions. Unique functions of BCs to scavenge reactive oxygen species (ROS) can resolve the redox imbalance due to the excessive generation of ROS. The ability of BCs to regulate the histone acetylation state contributes to the activation of transcription factors involved in immunity and metabolism against dietary stress. The protective properties of BCs are mainly ascribed to the roles of sirtuin 1 (SIRT1) and nuclear factor erythroid 2–related factor 2 (NRF2). As a histone deacetylase (HDAC), SIRT1 modulates the cellular redox balance and histone acetylation state by mediating ROS generation, regulating nicotinamide adenine dinucleotide (NAD+)/NADH ratio, and activating NRF2 in metabolic progression. In this study, the unique functions of BCs against diet-induced inflammation, oxidative stress, and metabolic dysfunction have been considered by focusing on the cellular redox balance and histone acetylation state. This work may provide evidence for the development of effective therapeutic agents from BCs.

## 1. Introduction

Bioactive compounds (BCs) are nutritional agents with diverse potentials exhibiting anti-inflammation, anti-oxidative stress, and anti-metabolic syndrome. The ability of BCs to modulate biological and physiological conditions may derive from their chemical and biological structures for favorable bioavailability and biochemical function [1,2]. The unique chemical structures of BCs enable them to effectively quench reactive oxygen species (ROS) for redox balance [2]. BCs have been demonstrated to control the expression of target genes and proteins via regulating epigenetic modifications, including the regulation of the histone acetylation state [3,4]. In redox signaling, inflammation, oxidative stress, and metabolism are considered electron-transfer processes reliant on the electron transport chain (ETC) [5,6]. The transfer of electrons to the final acceptor of the oxygen molecule is required for ATP synthesis. During several processes modulated by electron donors and acceptors, redox balance and stability are directly related to the metabolism and immune responses [7,8]. A sustainable and stable redox balance is essential for maintaining physiological pathways within the cells [7].

The function of BCs influences epigenetic changes that affect DNA repair and cell proliferation through the deacetylation of histones or non-histone proteins [9,10]. Depending on the redox environments, the post-translational modifications of histones are available in the N-terminal of core histone protein [10,11]. The alteration in histone acetylation is controlled by the opposing actions of histone acetyltransferases (HATs) and histone deacetylases (HDACs) [4,9]. The modulation of acetyl moieties can affect metabolic reprogramming and immune response. The hyperacetylation of histones has been reported to result in DNA damage, whereas hyperacetylated non-histone proteins increase ubiquitination [1,3]. Hypoacetylation with HDACs led to the exacerbation of diseases, while significant decreases in HDAC activity were found in patients with several diseases [11]. Histone deacetylation targeting the chromatin remodeling and its mobility resulted in the control of genomic binding for DNA recombination and access for histone-DNA interactions [12]. The coordination of epigenetic regulators is likely based on the redox state, as moieties’ accessing and binding features depend on the electric charge.

The dietary stress-induced change of the redox state causes a highly oxidative condition due to the excessive generation and accumulation of ROS, leading to oxidative stress, inflammation, and metabolic dysfunctions [2,13]. The oxidation state can be altered by supplementing BCs with the ability to scavenge ROS and electrons and activate sirtuin 1 (SIRT1) and nuclear factor erythroid 2–related factor 2 (NRF2), a master regulator of anti-oxidative responses [5,6]. Dietary stress, i.e., excessive alcohol consumption and a high-fat or high-glycemic diet, can cause alterations in glucose and lipid metabolism, leading to excessive fat accumulation and the production of ROS and inflammatory cytokines [14,15,16]. These metabolic signaling changes can alter cancers, liver diseases, diabetes, pancreatitis, and atherosclerosis [17,18,19]. The dietary stress triggers toll-like receptors (TLRs) and promotes the activation of nuclear factor-κB (NF-κB) or activator protein 1 (AP-1) by acetylation, increasing inflammation [14,16].

SIRT1 and NRF2 are considered prime targets for edible BCs [20,21,22]. SIRT1, a class III HDAC, is encoded in a nuclear-located gene with the largest N-terminal and C-terminal domains among its seven families [11,23]. The activation of SIRT1 adjusts the redox state to prevent inflammation, oxidative stress, and disorders by facilitating the positive feedback loop with NRF2 signaling [24]. SIRT1 is reported to promote the formation of the SIRT1-NF-κB or SIRT1-AP-1 axis and inactivate target transcription factors by deacetylation [25,26]. Cellular stress caused by excessive alcohol, high-fat, or high-glycemic diet may induce the inhibition of SIRT1 by the inhibitory protein, p53-acetylated peptides, forming a pseudo-substrate for SIRT1 inactivation [27,28]. However, supplementing BCs favors the interaction with SIRT1 to activate NAD+-regulatory elements in the C-terminal domain, resulting in the activation of SIRT1 [29,30,31]. In mouse liver, high-fat diet-induced p53 acetylation and transcriptional activity resulted in a lower level of SIRT1 [32,33]. However, the activation of SIRT1 by supplementing BCs repressed p53 activity by deacetylating it [27,28]. The deacetylation ability of SIRT1 activates farnesoid X Receptor (FXR) to promote the transcription of the small heterodimer partner (SHP), resulting in the repression of p53 and an increase in SIRT1 expression [34,35,36].

The present review summarized the current studies on the BCs exhibiting protective effects against inflammation, oxidative stress, and metabolic dysfunction by regulating the cellular redox balance and acetylation states of histone and non-histone proteins. Excessive alcohol, high-fat, or high-glycemic diet was considered dietary stress for the induction of the immune system and metabolic disorders. The results of this work can be utilized to provide a platform for developing therapeutic interventions by employing BCs.

## 2. Regulation of the Cellular Redox Balance and Histone Acetylation State

### 2.1. Regulation of Cellular Redox Balance

The cellular redox state is determined by the balance of equivalents representing reduced and oxidized ions [7,13]. The conformational alterations due to the shift of the redox state have been regarded to induce alterations in several physiological and pathological conditions [37]. The imbalance of the reduction toward the oxidation state due to the excessive generation of ROS and reactive nitrogen species (RNS) or the lack of antioxidants and their inactivation result in inflammation and oxidative stress [38].

The redox state of inflammatory cells and tissues is caused by the acetylation and oxidation state, as those states produce lots of electrons and free radicals, including ROS and RNS [13]. The energy production process, such as mitochondrial oxidative phosphorylation, generates metabolic intermediates and reduces equivalents. Electrons donated from nutrients are transferred to reducing equivalents such as NADH and to oxygen via electron carriers [39]. The electron transfer process is governed by redox states of the mitochondrial TCA cycle and ETC [7]. Dietary stress may disturb the normal transport process of electrons and reactive metabolites, leading to the imbalance of redox states for the induction of inflammation, oxidative stress, and metabolic dysfunction. The regulation of the cellular redox state is mediated by the cross-talk between NRF2 and NF-κB, the redox-sensitive transcription factors [40]. The antioxidant transcription factor NRF2 can modulate NF-κB activation by reducing ROS and inhibiting the degradation of the inhibitor of κB (IκB). Moreover, the pro-inflammatory transcription factor NF-κB can increase the recruitment of HDAC to the antioxidant response element (ARE) with the concomitant interference of NRF2 transcription [40,41]. The ability of BCs to lower the oxidation state can regulate detrimental oxidative conditions by scavenging electrons and ROS/RNS, activating NRF2 through SIRT1 activation as well as inhibiting NF-κB.

### 2.2. Regulation of Histone Acetylation State

**Figure 1:**
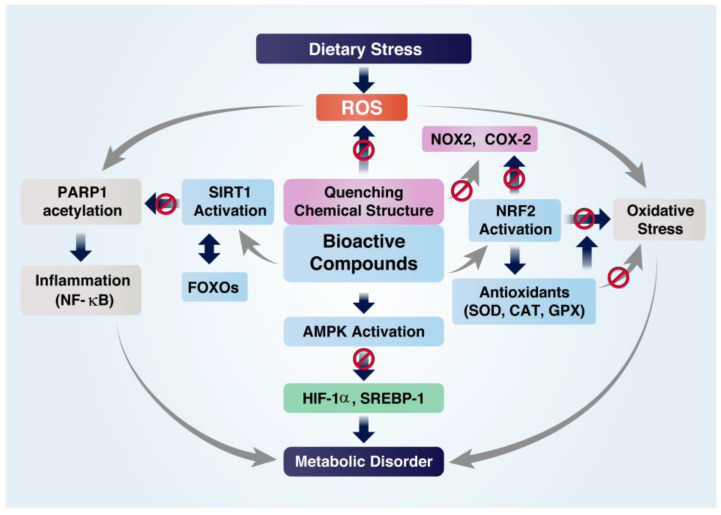
Role of bioactive compounds (BCs) in dietary stress-induced inflammation, oxidative stress, and metabolic disturbances. The role of BCs in dietary stress can be divided into two categories. One is directly scavenging or quenching reactive radicals, such as ROS, due to their conjugated double-bond chemical structure. The other role is to activate biological systems through the activation of the enzyme, SIRT1, and the transcription factor, NRF2. The function of SIRT1 is to activate forkhead box protein O (FOXOs) while inactivating NF-κB to suppress inflammation. BC-induced NRF2 activation prevents oxidative stress by activating antioxidants, including superoxide dismutase (SOD), catalase (CAT), and glutathione peroxidase (GPX). BCs prevent inflammation, oxidative stress, and metabolic disturbances through the activation of SIRT1 and NRF2.

Epigenetic alterations that regulate histone acetylation states have been found to dysregulate genes mediated by inflammation, oxidative stress, and metabolic disorders [3,11]. Controlling the histone acetylation can be an effective immune response and metabolic regulation against cellular and tissue dysfunction. Upon dietary stress, HATs are activated and acetylate lysine residues within lysine-rich amino-terminal tails of the core histones, such as H2A, H2B, H3, and H4 [10], which are easily modified [42]. Positively charged amino residues facilitate the tight structure with DNA, which is usually negatively charged [4,43]. The positive charge of the histone tail is neutralized by acetylation due to the covering of the negatively charged acetyl group (CH3CO-). Therefore, the tight structure between DNA and histone proteins is broken, allowing chromatin to access other transcription factors to meet the redox balance. However, the removal of the acetyl group from histone maintains the positive charge, enhancing the tight conformation between DNA and histone amino residues. In this circumstance, the chromatin mobility to access and bind to other transcription factors is condensed and inactivated [3,4] (Figure 1).

In response to dietary stress, including excessive alcohol, high-fat, or high-glucose diets, cells and tissues are acetylated, which may induce increased HDAC expression and the activation of TLRs [44]. As dietary stress is forced to generate lots of electrons, higher levels of ROS/RNS cause mitochondrial dysfunction and oxidative damage, i.e., lipid peroxidation in the oxidative and acetylated states [45,46]. The transition of histones to the acetylation state leads to the activation of NF-κB, a critical transcription factor in inflammation [25,26]. An imbalance in the histone acetylation state can be restored with BCs by deacetylation through the activation of their primary target, SIRT1. The post-translational deacetylation by SIRT1 has been controlled in response to cellular stresses [23,47], including excessive alcohol, high-fat, or high-glycemic diet.

**Figure 2:**
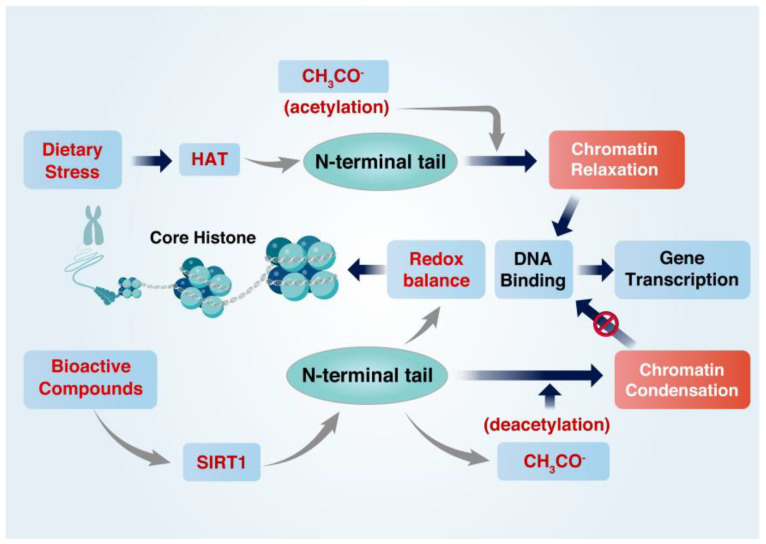
BCs activate SIRT1 for histone deacetylation, whereas dietary stress stimulates HAT for histone acetylation. Histones are the major components of chromatin and assemble with DNA to form nucleosomes. Dietary stress-induced HATs acetylate histone lysine residues, neutralizing the histone positive charge and enabling inflammatory gene transcription. However, BC-activated SIRT1 removes acetyl groups from histones, leading to chromatin condensation and the inhibition of inflammatory gene transcription.

The function of SIRT1 as a deacetylase is attributed to its various abilities to modulate redox states [24,48]. To inhibit inflammation and oxidative stress, SIRT1 represses NF-κB activation while activating NRF2, respectively (Figure 2).

SIRT1 has also been reported to activate the peroxisome proliferator-activated gamma coactivator 1 (PGC1), FOXO, and peroxisome proliferator-activated receptors (PPARs), leading to fatty acid oxidation and mobilization [23,49]. The deacetylation of FOXOs by SIRT1 activates SIRT1 transcription in a FOXO1-dependent manner, which facilitates the positive loop to maintain high levels of SIRT1 expression [50]. In the fasting condition, the increased glucagon levels and cAMP signaling modulate the localization of the cAMP response element-binding protein (CREB) to the nucleus and the binding with its co-regulator CREB-required transcription coactivator 2 (CRTC2) [51,52]. The binding of the CREB-CRTC2 complex to the promoter of SIRT1 induces the transcriptional activation of SIRT1. Interestingly, the activated SIRT1 deacetylates CRTC2 for degradation and represses gluconeogenesis, resulting in a metabolic shift from gluconeogenesis to fatty acid oxidation [51]. Another SIRT1 activation process is driven by decreased insulin-protein kinase B (Akt) signaling, which translocates FOXOs to the nucleus [49]. SIRT1 is activated by FOXO1 through the direct binding of the FOXO element to the SIRT1 promoter [50].

## 3. Diet-Induced Alterations of Redox Balance and Histone Acetylation State

### 3.1. Excessive Alcohol Use

Excessive alcohol use increases inflammation and oxidative stress in macrophages such as murine RAW 264.7 cells and bone marrow-derived macrophages (BMDMs) due to an increased acetylation state of NF-κB, an essential transcription factor for inflammatory responses [30,31]. Alcohol metabolites, such as the acetyl group and ROS, are generated during the cytosolic oxidation of ethanol to acetaldehyde and the subsequent mitochondrial oxidation to acetate [53,54]. Alcohol has been reported to acetylate the lysine residues of NF-κB, enhancing its nuclear translocation and thus increasing inflammatory gene expression [25,55]. Chronic alcohol consumption has been demonstrated to alter the intestinal microflora and its permeability, enhancing the release of lipopolysaccharide (LPS) [56,57]. LPS enters the liver and activates the resident macrophages, Kupffer cells, to release pro-inflammatory cytokines such as tumor necrosis factor α (TNFα). The LPS-induced TNFα, in turn, promotes liver injury and disease [58,59].

Excessive alcohol consumption significantly represses the transcription and activation of SIRT1. The alcohol or LPS-induced NADPH oxidase 2 (NOX2) enhances ROS generation [60,61], shifts the microenvironment to a hyperoxidative state, leads to oxidative stress, and suppresses SIRT1 [62,63]. As nicotinamide adenine dinucleotide (NAD+) is a cofactor of SIRT1, the profound reduction of NAD+ during alcohol metabolism may also affect the remarkable inactivation of SIRT1 [64,65]. NAD+ has been noted as an essential coenzyme for cellular energy metabolisms such as glycolysis, fatty acid oxidation, and alcohol metabolism concerning the NAD+/NADH redox balance [64,66]. Excessive alcohol consumption stimulates macrophages, a central component of the innate immune system, promoting inflammation and triggering the switch to aerobic glycolysis [67,68]. ROS accumulation, which leads to inflammation and oxidative stress, induces hypoxia-inducible factor 1α (HIF-1α) activation for the metabolic switch toward glycolysis in macrophages [68,69]. The activation of HIF-1α due to the acetylation by alcohol, in turn, increases the expression of downstream target genes for glycolysis, such as glucose transporter 1 (GLUT1) and hexokinase 1 (HK1) [31,68]. Interestingly, alcohol also alters mitochondrial respiration in that ethanol increases basal respiration, ATP production, and proton leak but decreases maximal respiration and spare respiratory capacity in macrophages [67,70].

BCs, such as astaxanthin and nicotinamide riboside, have been demonstrated to inhibit alcohol-induced inflammation, oxidative stress, and metabolic disorders due to their abilities to quench ROS and deacetylate the associated transcription factors through the activation of SIRT1 [30,31]. SIRT1 activates the antioxidant transcription factor NRF2 [30,53] and inhibits the activity of AP-1 through deacetylation, thereby repressing ROS-generating enzymes, such as NOX2 and cyclooxygenase-2 (COX-2) [48,71]. SIRT1 also inhibits NF-κB activity by suppressing its acetylation and nuclear translocation in alcohol-stimulated macrophages [30,72]. Notably, alcohol induces the phosphorylation of FOXO3 and inactivates the role of p53 binding. Furthermore, p53, the down-regulator of SIRT1, inhibits SIRT1 expression [24,47,73]. Alcohol-induced alterations in metabolic pathways, including glycolysis and mitochondrial respiration, have also been restored by SIRT1 due to its deacetylation [70,74]. BCs can also support the roles of adiponectin, an adipocyte-driven protein, to repress inflammation and alcoholic liver injuries by inhibiting LPS-induced TNFα production in Kupffer cells and murine macrophages through SIRT1 activation [60,75].

### 3.2. High-Fat Diet

A high-fat diet induces oxidative stress and disrupts the balance of the redox state, which affects inflammatory signaling [38]. Inflammatory pathways enhance ROS production and promote the redox imbalance toward oxidative stress [8,76]. Under high-fat diet conditions, there is a positive feedback loop between oxidative stress and inflammatory signaling. The induction of inflammatory genes is ascribed to the acetylation states caused by a high-fat diet since the acetylated state promotes the binding of chromatins to DNA for transcription [76,77,78]. As an adaptive response to a high-fat diet, an obese status emerges with an increased acetylation state and a concomitant decrease in HDAC1 [1,15]. A high-fat diet results in the hyperplasia of adipocytes, the uncontrolled expansion of adipocytes, and excessive fat accumulation in adipose tissue [79]. Excessive fat deposition inhibits the adipocyte activity to control lipid metabolism and energy expenditure, leading to inflammation, dyslipidemia, hypertension, cardiac and liver damage, and cancer [80,81]. The fat stored as triglycerides (TG) in white adipose tissue can be converted to brown-like tissue by inducing uncoupling protein 1 (UCP1) and simultaneously increasing energy expenditure [82]. The fat conversion leads to excessive oxygen consumption in mitochondria [83,84], consequently decreasing oxygen availability and resulting in reductive conditions.

A high-fat diet adversely affects the control of the hepatic energy metabolism. A high-fat diet has been demonstrated to induce insulin resistance with metabolic disorders in mice [18,52]. The ability of the liver to regenerate tissue in detoxification was negatively correlated with the protein level of HDAC1 in male rats [85]. In addition, liver proliferation was inhibited by CCAAT enhancer-binding protein α (C/EBPα) through HDAC1 in older male mice [85,86,87,88]. A high-fat diet also induced metabolic dysfunction in the liver through increased fatty acid synthase (FAS) and stearoyl-CoA desaturase and inhibited HDAC3, which is recruited to the promoters of the lipogenic genes during fasting [89]. Likewise, HAT-mediated acetylation in the liver increased FAS and concomitantly decreased HDAC9 expression in mice [89]. Increased HDAC3 in the liver in response to a high-fat diet reduced insulin sensitivity and lipogenesis [89]. The high-fat diet lowered the SIRT1 expression by decreasing CREB and increasing the recruitment of carbohydrate response-element binding protein (ChREBP) to bind to the SIRT1 promoter [51].

Furthermore, a high-fat diet promotes the interaction of proteasome activator subunit 3 (REGγ) with SIRT1, leading to the degradation of SIRT1 and increasing the acetylation state of autophagy proteins. The suppressed autophagy-dependent degradation of lipid droplets causes liver steatosis [90]. The high-fat feeding decreased SIRT1 protein levels in the white adipose tissues of mice by proteolytic cleavage [15]. These results indicate that a high-fat diet alters the acetylation state, leading to oxidation, which can be restored by supplementing BCs through the redox balance.

### 3.3. High-Glycemic Diet

Long-term high-glycemic diets lead to chronic metabolic disorders, resulting in high blood glucose, which causes multiple organ failure [91,92]. Hyperglycemia induces oxidative stress and reduces endothelial progenitor cells with the impairment of their ability to undergo endothelial differentiation and angiogenesis in response to vascular injuries [93,94]. HDAC2 has been reported to respond to hyperglycemia or diabetes-induced oxidative stress and has been implicated in high glucose-induced damage in endothelial progenitor cells [43,94]. In addition, hyperglycemia suppressed expressions of heme oxygenase 1 (HO-1) and SIRT1, which are restored by treatment with HDAC2 siRNA [94,95]. The repression or loss of HDAC2 attenuated the injuries in endothelial progenitor cells. HO-1 has been reported as an NRF2-regulated gene for the prevention of vascular inflammation and oxidative stress [96]. Taken together, a high-glycemic diet suppresses the HO-1/SIRT1 pathway and causes oxidative stress and vascular injuries with the induction of HDAC2.

Glucose and insulin levels are elevated in response to the chronic high-glycemic diet, which is responsible for the inactivation of pancreatic β-cells. The pancreatic β-cells have been demonstrated to sense glucose levels by interacting with pancreatic duodenal homeobox 1 (PDX1) or HIF-1α [97,98]. At high glucose levels, PDX1 is involved in acetylating the promoter of genes related to insulin secretion by activating p300. However, PDX1 is mediated with HDAC1 and HDAC2 in relatively low plasma glucose levels, leading to the repression of insulin gene transcription by deacetylation [99,100]. The activity of HIF-1α is known to be repressed in high glucose levels since methylglyoxal, a reduced derivative of pyruvic acid generated during glycolytic pathways, inhibits HIF-1α transcription by suppressing the acetylation with p300 [101]. High glucose levels in adipocytes result in c-Jun N-terminal kinase (JNK) activation, leading to SIRT1 degradation [102]. In contrast, glucose starvation inhibits the interaction of REGγ, a proteasome activator, with SIRT1 by the 5′ AMP-activated protein kinase (AMPK), leading to an increase in SIRT1 [90]. Therefore, BCs may need to activate SIRT1 and HO-1 to regulate the glucose metabolism to prevent hyperglycemia-induced damage and disease.

## 4. Protective Roles of Bioactive Compounds (BCs)

Relatively small quantities of chemicals that are present in natural sources, i.e., plant, animal, and algae, and that have health benefits are considered BCs [103]. BCs are classified by their chemical structure and biochemical functions. Examples of BCs include carotenoids, bioactive fatty acids and peptides, polyphenols, glucosinolates, triterpene, and phytosterols [103,104]. Gut microbiota, essential for the modulation of intestine functions such as the fermentation and absorption of short-chain fatty acids, interact with BCs [57,105]. The biodiversity and activity of gut microbiota depend on various pathophysiological events, including immune function and energy metabolism [56,106]. Gut dysbiosis induced by the prolonged intake of alcohol, high-fat, or high-glycemic diet can increase bacterial lipopolysaccharide, oxidative stress, pro-inflammatory cytokines, intestinal inflammation, and intestinal permeability [107,108]. The relationship between dietary BCs, gut microbiota, and metabolism has demonstrated that gut dysbiosis, the imbalance of gut microbiota associated with an unhealthy outcome, can be attenuated by supplementing BCs.

### 4.1. Inhibition of Inflammation

Inflammation is one of the responses triggered by the innate immune response. ROS and redox-sensitive transcription factor NF-κB are induced in response to the inflammatory stimulus, such as dietary stress [109]. The dietary stress also elevates the gut-derived LPS production and translocation into the systemic circulation, triggering TLR4 and NF-κB activation and enhancing pro-inflammation cytokines [14]. The production of pro-inflammatory cytokines such as TNFα and interleukins (ILs) affects the activation of NF-κB and mitogen-activated protein kinase (MAPK) cascades. The MAPK signaling cascades, including extracellular signal-regulated kinases (ERKs), JNKs, and p38, are activated by transforming growth factor β-activated kinase 1 (TAK1). TLRs activate this signaling by recognizing changes in various molecular patterns and recruiting suitable adaptors to stimulate the downstream signaling pathways and generate pro-inflammatory cytokines. In addition, NF-κB induces the production of adhesion molecules to facilitate phagocyte infiltration.

By inhibiting pro-inflammatory cytokines effectively, other downstream inflammatory pathways and cascades are blocked [110]. One of the critical roles of BCs in the immune response is maintaining balanced pro- and anti-inflammatory signals to avoid overreaction leading to tissue damage and disease [11,68]. The selective functions of BCs, such as astaxanthin, nicotinamide riboside, and resveratrol, come from their ability to activate SIRT1 [111,112]. Typical roles of SIRT1 in regulating inflammation, metabolic disorder, apoptosis, and cell cycle regulation have been discovered [23,113]. SIRT1 inhibits NF-κB transcriptional activity by deacetylating its subunit, RelA/p65, and it suppresses NF-κB-mediated TNFα, inhibiting inflammation [25,26]. SIRT1 represses the p300/CREB binding protein (p300/CBP), a central acetyltransferase, and inhibits p65 acetylation, which is essential for turning off NF-κB-mediated gene expression [72]. In addition, the p300/CBP is known to acetylate poly (ADP-ribose) polymerase 1 (PARP1) for NF-κB activation [113,114].

### 4.2. Inhibition of Oxidative Stress

Excessive ROS production is triggered by the innate immune system upon dietary stimulation. ROS, such as hydroxyl radical (HO•), superoxide anion (O_2_^−^), and hydrogen peroxide (H_2_O_2_), are composed of radical and non-radical oxygen species formed by the partial reduction of oxygen [46,115]. In response to cellular damage by ROS, endogenous antioxidants such as catalase and superoxide dismutase are activated as cellular and tissue defenses [37,116]. Excessive ROS production beyond the antioxidant defense capacity disrupts the redox balance and defense systems, leading to oxidative stress and various pathogenic pathways, including DNA damage, mitochondrial dysfunctions, and endoplasmic reticulum stress [117]. Exogenous antioxidants may be required to regulate the excessive ROS and circumvent chronic cellular inflammation. The effective inhibition of ROS-induced oxidative stress can proceed in two ways. One is scavenging ROS and free radicals generated in the system, and the other is blocking the pathways that derive oxidative stress [6,116]. BCs are suitable agents to provide roles against oxidative stress in both ways. The chemical structure of BCs is highly effective for scavenging generated ROS and free radicals and balancing redox reactions [118,119]. BCs activate NRF2 to regulate ROS production with its downstream target genes, HO-1 and NAD(P)H quinone dehydrogenase 1 (NQO1) [96,120]. In tumor cells, the suppression of NRF2 increases ROS production, inducing autophagy and cell death by AMPK activation [121,122]. Furthermore, the activation of SIRT1 by supplementing BCs modulates p53 and FOXO3a by deacetylation [123,124], leading to the activation of antioxidant enzymes. The oxidative stress induced by D-galactose in human trophoblast HTR8/SVneo cells can be prevented by forming a SIRT1/FOXO3a/ROS signaling pathway to scavenge ROS [125]. SIRT1 also exhibits antioxidant properties by deacetylating p53 [126,127] and NRF2 inhibitors [86,87,88]. Diabetic nephropathy driven by p53 acetylation is inhibited through the deacetylation of p53 by SIRT1 [20,47]. SIRT1 activates NRF2 by inhibiting p53 through deacetylation, formulating the SIRT1/p53/NRF2 pathway to regulate the pathogenesis of diabetic nephropathy [20,47]. Evidence suggests that NRF2 inhibits ROS production with SIRT1, regulating redox balance and inducing antioxidants for ROS scavenging.

NRF2 binds to Kelch-like ECH-associated protein 1 (Keap1) in the cytoplasm in normal conditions. Binding Keap1 to Cullin3, a scaffolding protein, results in the degradation of NRF2 through ubiquitination [128,129,130]. Electrophiles such as sulforaphane and tert-butylhydroquinone interfere with the proteasome-mediated degradation of NRF2 by binding to Keap1 and altering the conformation of ligase that facilitates the escape of NRF2 from proteasomal degradation [77,131,132]. Upon the stimulation of oxidative stress, NRF2 is dissociated from Keap1, translocated to the nucleus, and bound to the ARE [88,133]. Under relatively low oxidative stress conditions, NRF2 is acetylated by various stimuli and then activated as a master regulator for antioxidant genes, including catalase, glutathione peroxidase, and superoxide dismutase [6]. Acetylated NRF2 by p300/CBP likely binds to DNA in the promoter region of genes encoding detoxification enzymes [134]. However, the hyper-acetylation of NRF2 in highly oxidative conditions fails to exert its unique functions due to its high redox sensitivity [6,78]. Indeed, the activity of NRF2 is decreased at high levels of oxidative stress, such as chronic obstructive pulmonary disease [78]. The inhibition of HDACs at such highly oxidative conditions increases the acetylation state of NRF2, decreasing its stability and activity, leading to impaired antioxidant properties and the concomitant reduction of NRF2-regulated genes [95,135]. This impairment is abrogated by lowering the acetylation state of NRF2 using HDACs, particularly HDAC2 [95]. However, the deacetylation of NRF2 by HDACs in normal or relatively low oxidative conditions reduces the NRF2 activity and its downstream genes [135]. The evidence indicates that the functions of NRF2 as an antioxidant may depend on its acetylation state, balancing delicately between the production and the elimination of ROS.

### 4.3. Inhibition of Metabolic Disorders

Dietary stress, such as excessive alcohol, high-fat, or high-glycemic diet, activates the innate immune system, including the reprogramming of energy metabolism favoring aerobic glycolysis in macrophages [136]. The altered energy metabolism in response to oxidative stress or inflammation causes rapid ATP production [69,137] and excessive mitochondrial ROS generation, affecting the electron transport chain related to mitochondrial respiration [39,116], consequently resulting in redox imbalance. The dietary stimulation can reduce the cytosolic NAD+/NADH ratio, leading to mitochondrial dysfunction with increased ATP production and proton leakage [31,39]. The changes in energy metabolism and mitochondrial dysfunction can be reversed by supplementing BCs with SIRT1 activation. SIRT1 has been reported to suppress the diet-induced HIF-1α activation and inhibit aerobic glycolysis by deacetylation. It has also been known to enhance PGC1α-mediated genes and promote mitochondrial proliferation and oxidative phosphorylation [46,138]. When oxidative stress emerges, high glucose levels due to metabolic disturbances can damage cells. The increased glucose may activate the function of SIRT1 on the NRF2-ARE signaling pathway as an adaptive pathway to avoid cellular damage [5,133]. The SIRT1-mediated adaptive pathway has similar effects on the metabolism by supplementing BCs [139]. SIRT1 also modulates mitochondrial biogenesis and oxidative metabolism to protect against metabolic disturbance by regulating mitochondrial contents and β-oxidation [64,65].

In lipid metabolism, the activation of SIRT1 promotes the liver kinase B1 (LKB1) and AMPK, constituting the SIRT1/LKB1/AMPK axis [140,141]. The SIRT1-driven axis plays a critical role in hepatic fatty acid oxidation through the deacetylation or phosphorylation of related transcription factors, including FOXO1 and sterol regulatory element-binding protein 1 (SREBP-1) [50,98,140]. Activated FOXO1 induces adipose triglyceride lipase (ATGL), the rate-limiting enzyme for lipolysis, while SREBP-1 promotes lipogenesis by inducing FAS [46,142]. The activation of AMPK by SIRT1 upregulates FOXO1 but inhibits the activity of SREBP-1 to prevent lipid accumulation in high fat-fed mice and HepG2 cells [143].

## 5. Roles of BCs in Regulating Cellular Redox Balance and Histone Acetylation State

### 5.1. Astaxanthin

Astaxanthin (ASTX), abundant in marine animals including krill, shrimp, crawfish, Asteroidea crabs, and lobster, is a lipid-soluble xanthophyll carotenoid with anti-inflammatory and antioxidant effects [144]. Due to its chemical structure of the conjugated double bond, ASTX is highly effective for quenching or scavenging ROS, attenuating NOX2 and COX-2, which are enzymatic sources for the production of ROS [119,145]. Moreover, ASTX can easily pass through the membrane bilayer due to its configuration with an oxo functional group without toxic side effects [109,118]. The unique features of ASTX contribute to its potential for various biological, biochemical, and physiological activities, including the activation of SIRT1 and NRF2 [30,146].

The anti-inflammatory and antioxidant properties of ASTX have been shown in macrophages stimulated with lipopolysaccharide, hydrogen peroxide, or alcohol [146,147], apolipoprotein E-knockout mice [148], or hepatic stellate cells [149]. ASTX has been shown to protect against diabetes, liver diseases, cancers, cardiovascular diseases, and pancreatitis by regulating NF-κB and NRF2 [150,151]. The ability of ASTX to exert anti-inflammatory and antioxidant properties in ethanol-stimulated macrophages may be due to the activation of SIRT1 and NRF2, in addition to enhancing cellular NAD+ levels [30]. External stimulation, such as excessive alcohol exposure, elevated NOX2 expression, and induced cellular oxidative stress, thereby rushing macrophages to produce large amounts of ROS [152,153]. ASTX supports NAD+, a cofactor to activate SIRT1, which facilitates FOXO3 binding to p53 to suppress p53 and activates itself. Furthermore, the ability of ASTX to recover the reduction state by scavenging ROS contributes to the regeneration of NAD+ from NADH, leading to the activation of SIRT1.

Under the dietary stress condition, NF-κB lysine residues are acetylated to allow the release from IκB, enhancing nuclear translocation and DNA binding, resulting in the transcription of its target inflammatory genes [43,154]. ASTX inhibits the nuclear translocation of NF-κB by deacetylating it [25,146] and counteracts NF-κB by inducing its corepressors, such as the transducin-like enhancer of split 1 (TLE1) through SIRT1 activation [155]. SIRT1 can also inactivate the coactivators of NF-κB, such as p300/CBP, to not acetylate p65 NF-κB in the nucleus [72]. Upon stimulation, p300/CBP acetylates PARP1 and directly interacts with p300 to activate NF-κB, which is prevented by SIRT1 through PARP1 deacetylation [113,114]. The function of ASTX may be due to the ability of SIRT1 to deacetylate NF-κB and inhibit its activity by preventing its nuclear translocation and modulating interaction with transcriptional repressors or coactivators. ASTX reduced the hepatic lipid accumulation from a high-fat diet by activating PPARα and regressing PPARγ and Akt in mice [109,156]. The inhibition of Akt activity by ASTX causes a decrease in the nuclear translocation of SREBP-1 and thus reduces hepatic lipogenesis. ASTX may also induce hepatic autophagy by activating PPARα and inhibiting PPARγ and Akt [151]. The clearance of lipids by ASTX supplementation may be ascribed to the ability of SIRT1 to deacetylate the functional proteins involved in each pathway [30].

### 5.2. Butyrate

Butyrate, found in butter and cheese, is a short-chain fatty acid formed from dietary fibers by colonic microbiota through fermentation [157,158,159]. Butyrate formation is affected by the gut microbiota’s structure, diversity, and composition, which is mediated by pathological routes such as inflammatory and metabolic diseases, diabetes, and atherosclerosis [160,161]. Butyrate can be an anti-inflammatory and anti-carcinogenic agent by mediating drug, energy metabolism, and intestinal homeostasis [162]. Colonocytes mainly consume butyrate [159], and small amounts reach the portal vein and systemic circulation to deliver bacteria-formed butyrate to the liver and involve the hepatic metabolism [157,163]. Butyrate inhibits intestinal inflammation and colorectal cancer by preventing HDACs, depending on the cellular energy state [105]. Butyrate inhibits cell proliferation in energy-rich cells but is used for energy in energy-deficient cells [158]. In the liver, butyrate inhibits HDAC3 activity and induces fibroblast growth factor 21 (FGF21) expression to increase fatty acid oxidation and ketogenesis [164], as well as PGC1α expression and TCA cycle flux [1]. In HepG2-C3 and primary human hepatocytes, butyrate activates the aryl hydrocarbon receptor, a specific nuclear receptor that regulates cytochrome p450 (CYP) enzymes and their target genes in drug metabolism [157]. The potential effects of butyrate on drug metabolism are likely due to its epigenetic action of inhibiting HDACs in the liver [157,165]. In brown adipose tissue, butyrate increases PGC1α and UCP1, indicating that butyrate increases thermogenesis along with energy dissipation and improves glucose tolerance and insulin levels in high-fat-fed mice [156]. Butyrate exhibits protective properties against glucose intolerance and insulin resistance induced by a high-fat diet by inhibiting HDAC activity [160]. In addition, butyrate activates NRF2 in a small intestine epithelial cell line (IEC-6 cells) and a human colorectal adenocarcinoma cell line (HT-29 cells) by inhibiting HDAC activity [166].

Recent studies have shown that butyrate inhibits the activation and proliferation of T-cell receptor (TCR)-driven gut human lamina propria CD4 T cells and thus prevents inflammatory cytokine production through inhibiting HDACs [167], as evidenced in murine splenic and human peripheral blood CD4 T cells [168,169,170]. Interestingly, the ability of butyrate to enhance histone acetylation is possibly associated with the G protein-coupled receptor (GPCR) signaling in the inactivation of intestinal lamina propria CD4 T cells, altering cellular metabolism [167] and inducing PPARγ signaling pathways [158].

### 5.3. Polyphenols

#### 5.3.1. Curcumin

Curcumin, found in the Indian spice turmeric, is a yellow-color polyphenol with potent antioxidant, anti-inflammatory, and anticancer properties [171,172,173]. Curcumin has been demonstrated to restore the impaired antioxidant function of NRF2 as a potential treatment for obesity and diabetes [174,175]. It inhibits skin tumorigenesis in mice [176] by preventing COX-2 expression by repressing NF-κB activity in mouse skin [177]. Curcumin inhibits the acetylation of histone and non-histone proteins as a HAT inhibitor [172]. It inhibited p300/CBP by inducing apoptosis in cancer cells through p53 signaling [172,178]. Curcumin has been understood to prevent the diet-induced elevation of the acetylation state, leading to NF-κB inactivation and the inhibition of inflammation [179,180]. Under high glucose levels, curcumin suppressed NF-κB activity and the production of IL-6 and TNFα [181]. Elevated blood glucose levels stimulate inflammatory signaling through the NF-κB pathway, indicating a close relationship between redox imbalance and inflammation [16]. Curcumin supplementation reversed the high-fat diet-induced insulin resistance and increased HAT levels due to its ability to adjust the balance between HATs and HDACs [180]. The usage of curcumin has been extended to protect against diabetes by inhibiting the endothelial nitric oxide synthase and transforming growth factor beta 1 (TGF-β1) in the kidney of rats through inhibiting p300/CBP and, thus, the repression of NF-κB [182].

Curcumin has been mediated to compensate for the deficiency of NRF2 antioxidant action by restoring the redox balance, which contributes to inhibiting inflammatory signaling [177,183]. In oxidative or insulin-resistant conditions due to a high-fat diet or diabetes, the ability of curcumin to repress Keap1 expression during inflammatory signaling leads to the upregulation of NRF2 [173]. It induced the transcription and translation of HO-1 in mouse skin in vivo and cultured murine epidermal cells through NRF2 activation [177,183]. The ability of curcumin may derive from its chemical structure of two electrophilic α and β-unsaturated carbonyl groups. The modification of Keap1 cysteine by this electrophilic BC is recognized to inhibit Keap1 ability, which suppresses NRF2 activation [183]. Curcumin stabilizes NRF2 by blocking 26S proteasomal degradation and inhibits the Cullin3-E3 ubiquitin ligase (RBX1) complex for NRF2 ubiquitination by modifying cysteine residue in Keap1, C151. Therefore, the binding capacity of Keap1 to NRF2 altered by curcumin can lead to the nuclear translocation of NRF2 [131,184].

Despite its benefits in suppressing high-fat-induced metabolic disorders and inflammation, curcumin remains a bioavailability challenge due to its limited absorption. The intestine is a barrier to the application of curcumin [171]. Encapsulation is one way to circumvent curcumin delivery to enhance its bioavailability and potential health benefits for humans [185].

#### 5.3.2. Epigallocatechin-3-Gallate

Epigallocatechin-3-gallate (EGCG), a major flavonoid compound of green tea, exhibits health-beneficial properties, including antifibrotic, antioxidant, anti-cancer, and antihypertensive properties [186,187,188]. Interestingly, EGCG exhibits a potent inhibitor of HAT activity, whereas green tea polyphenol shows an HDAC inhibitor in human prostate cancer LNCaP and PC3 cells [189]. Since green tea polyphenol contains the major polyphenolic compounds catechins, the effects of EGCG and green tea polyphenol on HAT and HDAC may be different. The ability of EGCG to inactivate HAT is due to its interaction with HAT enzymes rather than its reduction [189,190]. The ability to reduce HAT activity prevents obesity-induced inflammation, weight gain, and elevated blood glucose levels in male C57BL/6 mice [191] and female Sprague Dawley rats [192]. The inhibitory effects of EGCG on HAT activity prevent NF-κB acetylation and inhibit its activity with p300 at the inflammatory cytokine promoters such as IL-6 [190,193]. High-dose supplementation of EGCG, however, can cause hepatotoxicity owing to its pro-oxidant effects [194]. These pieces of evidence demonstrate that the protective effects of EGCG against obesity induced inflammation and metabolic disorders are dose-response phenomena by controlling the acetylation state via the inhibition of HAT activity.

#### 5.3.3. Resveratrol

Resveratrol, enriched in grapes, red wine, and berries, is a natural polyphenolic compound exhibiting protective effects such as anti-inflammation, anti-oxidative stress, and antiviral and antibacterial immunity [111,112,195]. The properties of resveratrol can be divided into two categories. One is due to its ability to regulate the redox state, and the other is for controlling the histone acetylation state. The former property corresponds to its chemical structure with conjugated double bonds [112,195]. As resveratrol scavenges free radicals, including ROS, the diet-induced oxidation state in the microenvironment can change to the reduction state with a concomitant decrease in oxidative stress [111]. The latter property is related to its ability to activate HDACs, such as SIRT1, a key enzyme for the protective roles of resveratrol [21]. Indeed, two pathways of resveratrol have been shown to suppress inflammation. In a human colon-derived myofibroblast cell, resveratrol reduced TNFα-induced ROS production and enhanced SIRT1 activation [196]. Additionally, it reduced the TNFα-induced activation of intercellular adhesion molecule-1 (ICAM-1), which causes inflammation without the involvement of SIRT1 using EX-527, SIRT1 inhibitor, and knockdown of SIRT1 [196]. In addition, SIRT1 is known to repress NF-κB by deacetylating the RelA/p65 subunit at lysine 310 [21,196,197]. Collectively, resveratrol inhibited TNFα-induced inflammation through redox- as well as acetylation-regulated pathways.

The protective function of resveratrol against lipid peroxidation in cell membranes and DNA damage may be due to its property of scavenging or quenching ROS [111]. The therapeutic effects of resveratrol have been found in metabolic and cardiovascular disorders, cancers, tuberculosis, and other age-dependent diseases [21,111,112]. The multiple effects of resveratrol are attributed to its ability to activate SIRT1 [198,199]. In Mycobacterium tuberculosis-infected mice, resveratrol inhibits TAK1, preventing phosphorylation and ubiquitination from inactivating associated signaling such as MAPK and NF-κB pathways through SIRT1 activation [200]. In peritoneal macrophages of mice and patients with active tuberculosis, SIRT1 expression is influenced by the TLR2-p38 pathway. In paraquat-induced lung-injured mice, resveratrol inhibits oxidative stress and lung injury through potent signaling between SIRT1 and NRF2. Resveratrol upregulated SIRT1 expression in mice lung tissue exposed to paraquat and attenuated oxidative stress and lung injury by activating NRF2 [21]. The relationship between SIRT1 and NRF2 seems complicated in resveratrol therapy. SIRT1 activates NRF2 by reducing its acetylation state, which is elevated by an oxidative stress response [135]. The anti-oxidant properties of NRF2 may depend on its acetylation state through the delicate balance of acetylation and deacetylation. SIRT1, activated by resveratrol, has been reported to promote nuclear translocation, DNA binding, and the transcriptional activity of NRF2, leading to NRF2-mediated gene expression [77,201,202]. Evidence has shown that increasing the acetylation state of NRF2 reduces the stability of NRF2, which impairs its antioxidant properties [95]. Resveratrol deacetylates SIRT1 and enhances the stability of NRF2 in cells or tissues, as oxidative stress is accompanied by its acetylation state.

Resveratrol has been demonstrated to reduce the expression of fibronectin and TGF-β1 induced by advanced glycation end products in the kidney of diabetic rats and mesangial cells [201,202]. The potency of resveratrol was due to its remarkable antioxidant properties through the NRF2/ARE pathway activated by SIRT1 [201,202]. Metabolic stimuli, such as high levels of glucose owing to metabolic disorders, may encourage cells to adopt adaptive signaling pathways to mitigate damage. Cells can adapt the NRF2/ARE antioxidant pathway in response to metabolic stimuli as an adaptively activated pathway [5,133]. The supplementation of resveratrol requires the role of SIRT1 as a deacetylase for the adaptive activation pathway [97].

### 5.4. Nicotinamide Riboside

Nicotinamide riboside (NR), a natural precursor of NAD+, comprises pro-vitamin B3 in cow milk [203,204]. NR activates SIRT1 by supplying the cofactor, NAD+. NR supports NAD+, a critical modulator of cellular processes, including metabolic pathways, and promotes SIRT1 expression [55,66]. NR exerted anti-inflammation and anti-oxidative effects and repressed metabolic disturbances in ethanol-exposed macrophages through SIRT1 activation [31,65,205]. NR is converted to bioavailable NAD+ by nicotinamide riboside kinase (NRK) and nicotinamide mononucleotide adenylyltransferase (NMNAT) or the NAD+ salvage pathway by converting nicotinamide [64,205], which leads to activating SIRT1.

Inflammatory macrophages may shift the glucose metabolism toward aerobic glycolysis [67,68] based on the innate immune response [136]. NR, however, regulates energy metabolism, including glycolysis and mitochondrial respiration, through SIRT1 activation [31,48]. NR inactivated HIF-1α by deacetylation [206] and repressed its downstream genes, such as GLUT1, pyruvate dehydrogenase kinase 1 (PDK1), and lactate dehydrogenase α (LDHα), which were increased in macrophages upon ethanol exposure [31,53]. The capacity of NR to mediate energy metabolism derives primarily from its role in SIRT1 activation. The induction of HIF-1α is enhanced by lactate, resulting in the upregulation of glycolysis and the repression of the TCA cycle by activating PDK1 [207,208,209]. Pyruvate, the final product of glycolysis, is converted to acetyl-CoA in mitochondria by pyruvate dehydrogenase (PDH), whose activity is suppressed by PDK1 via phosphorylation [210,211]. Considering the repression of GLUT1, PDK1, and LDHα expression by SIRT1, the metabolic disturbances induced by excessive alcohol consumption can be reprogrammed by NR through SIRT1 activation and its deacetylation capacity [31,48]. NR also regulates mitochondria biogenesis in hepatocytes through SIRT1′s ability to deacetylate PGC1α [48,52].

### 5.5. Sulforaphane

Sulforaphane (SFN), an isothiocyanate compound found in cruciferous vegetables such as broccoli, kale, cauliflower, brussels sprouts, and cabbage, can induce phase II detoxifying/antioxidant enzymes by activating NRF2 [212]. The chemical structure of SFN, a β-D-thioglucose group linked to a sulfonated aldoxime moiety, is effective in treating cancers in the liver, breast, ovary, thyroid, and lung [212,213,214]. Under oxidative stress conditions, SFN activates NRF2 to bind to ARE in the promoters of antioxidant enzymes, exhibiting antioxidant and anti-inflammatory properties [214]. SFN suppresses cancer cells such as HCT116 colon cells and human embryonic kidney 293 cells by simultaneously inhibiting the activities of HDACs such as HDAC2 and HDAC3 [213,215]. The inactivation of HDACs in cancer cells is likely due to increased acetylation in histone H3 and H4 mediated by SFN metabolites, including SFN-N-acetyl-cysteine [216,217,218]. SFN has also been reported to increase the levels of ROS initiating the apoptotic pathways [2] while it decreases ROS levels in several cancer cells already in the apoptotic process [214]. The evidence indicates that the effects of SFN on ROS production are dose-and time-dependent and are involved in preventing cancer cell proliferation through the activation of caspase 3 and 8 [213,219]. SFN can suppress pro-survival pathways such as NF-κB activation [220,221] and induce caspase-mediated cell death by repressing HDAC6 activity and increasing the acetylation state of p21 and Bcl-2-associated X protein (BAX) promoters [214,219]. The inhibition of HDAC6 by SFN in prostate cancer cells increases the acetylation state of heat shock protein 90 (Hsp90) and decreases the expression of the androgen receptor (AR) [222].

SFN enhances NRF2 expression in prostate cancer TRAMP C1 cells via demethylation or acetylation [223]. SFN was demonstrated to act as an HDAC inhibitor since it decreased HDAC1, 4, 5, and 7 at the protein level in cancer cells [213,223]. It inhibited class I HDACs, such as HDAC2, and class IIa HDACs such as HDAC4, 5, 7, and 9, isoforms in 3T3-L1 adipocytes [215]. In TRAMP C1 cells, SFN increased the acetylated histone 3 levels, which elevated binding DNA to the NRF2 promoter, inducing its expression [223]. SFN also inhibits adipocyte differentiation, which leads to insulin resistance and type 2 diabetes due to triglyceride storage disorders [79,218]. HDACs were reported to be negatively correlated to adipocyte differentiation as the activities of HDAC1, 2, 5, and 9 are downregulated during adipocyte differentiation [224]. The repression of HDACs results in hyperacetylation in the promoters of adipogenic genes, including PPARγ, fatty acid-binding protein 4 (FABP4), and FAS, and p300 at the promoter of C/EBPα, respectively [225,226]. Additionally, the suppression of HDACs by inhibitors appeared to inhibit the differentiation of adipocytes [227,228]. The effects of SFN on adipocyte differentiation through the activity of HDACs need to be elucidated in future studies. Recently, SFN has been elicited to prevent cell cycle arrest and apoptosis in oral cancer cells by inhibiting HDACs [212,213]. The function of SFN has been shown to suppress cancer cell proliferation and the activity of HDACs, especially under oxidative stress with increased ROS or decreased mitochondrial membrane potential [212,218]. The anticancer properties of SFN are due to its potential to inhibit HDACs activity, cell cycle arrest, and apoptosis, as well as antioxidants and anti-inflammatory potential through activating NRF2 and repressing NF-κB.

### 5.6. Ginsenoside

Ginseng, a well-known medicinal herb, has been identified to contain bioactive ingredients, including ginsenosides, polysaccharides, steroids, and flavonoids. Ginsenosides are the primary pharmacologically active ingredients in ginseng [229,230]. More than 250 identified ginsenosides were classified into three categories [231]. However, the effects of total ginsenosides extracts (TGS) are discussed in this review. TGSs have been shown to affect oxidative stress, inflammation, and diabetes and regulate autophagic and apoptotic properties by mediating glucose and lipid metabolism [229,232]. TGS enhances mitochondrial energy metabolism by mediating mitochondrial oxygen consumption, respiration, and ATP production in several cell lines, such as cardiomyocytes, skeletal myoblasts, and vascular endothelial and epithelial cells [233,234]. The beneficial effects of TGS on cardiomyocytes and neurons are ascribed to its ability to activate SIRT1 to promote mitochondrial biosynthesis through the SIRT1/PGC1α axis and prevent brain injury and ischemic heart [138,235]. Considering that PGC1α binds and coactivates NRF2 in the mitochondrial transcription factor A (TFAM) promoter, TGS is regarded to activate NRF2 to improve mitochondrial function [235,236]. Cumulative evidence suggests that TGS protects brain ischemia by modulating TLR4/MyD88 and SIRT1 pathways associated with NRF2/ARE pathways [237,238].

Ginsenosides inhibit inflammation by suppressing inflammatory signaling pathways such as NF-κB and producing pro-inflammatory cytokines [239,240]. They repress MAPK phosphorylation induced by LPS, promote the activation of CREB [241], and block the activation of JAK2/signal transducer and the activator of transcription 3 (STAT3) in LPS-induced RAW 264.7 macrophages [230]. Ginsenosides repress NF-κB subunit p65, COX-2, ERK, JNK, and MAPK p38 in acute liver injury by exploiting their anti-inflammatory capabilities via NF-κB and MAPK pathways [242]. Ginsenosides also inhibit liver fibrosis by regulating TNFα, IL-1β, IL-6, and caspase-1 in response to the JNK- and p38/ERK pathways. The protective effects on liver injury are associated with the SIRT1/NRF2/NF-κB signaling pathway [243,244]. The function of ginsenosides to inhibit cell surface receptors such as TLR4 and its downstream targets, NF-κB and MAPK, leads to liver protection from LPS-induced acute injury in mice [245]. Ginseng is effective in treating colon, lung, and breast cancers [246], and its combination therapy with antitumor drugs has been reported to improve therapeutic efficacy [247,248]. The antitumor effects of ginseng are partly attributed to its metabolites, 20s-protopanaxadiol, by repressing the phosphorylation of the epidermal growth factor (EGF) and its receptor (EGFR) and inhibiting the activation of ERK1/2, p38, and c-JNK [249]. Ginsenosides have been found to inhibit cancer cell growth by altering the tumor microenvironment and enhancing antitumor immunity, with the selective inhibition of the ROS/ERK pathway [250,251] and blocking the EGF-EGFR-ERK1/2 or ERK-Akt pathway [252,253]. Antidiabetic effects of ginsenosides have been discovered through their ability to reduce gluconeogenesis while promoting glucose transport and insulin production. Furthermore, their hepatoprotective and anti-inflammatory potential as antioxidants or immunosuppressants can help their antidiabetic effects partly through the STAT5-PPARγ, AMPK-JNK, or NF-κB pathways [254,255]. Collectively, the beneficial effects of ginseng or ginsenosides can be ascribed at least partly to their ability to regulate the cellular redox balance or histone acetylation state through the activation of SIRT1 and NRF2 and their downstream genes.

## 6. Conclusions

BCs inhibit inflammation, oxidative stress, and metabolic disorders by regulating dietary stress-altered oxidative microenvironments. The role of BCs is achieved by controlling the cellular redox balance and histone acetylation state in response to biological conditions. The selective functions of BCs can be classified into two categories. One facilitates the reductive microenvironment from an oxidation state by quenching or scavenging free radicals, such as ROS. The other regulates the activity of transcription factors related to the immune system and metabolism. The former function is attributed to their chemical structures, such as conjugated double bonds, related to bioavailability, while the latter has the ability to activate the protective pathways by the induction of SIRT1 or NRF2 and its target genes. The function of BCs in adjusting cellular redox balance and the histone acetylation state to interact with a metabolic balance is highly complicated, as several crosstalks among enzymes and proteins are involved. Potential mechanisms of action by BCs to modulate cellular redox balance and histone acetylation state are summarized in Table 1. BCs exhibit multiple complex properties, enabling the opposite function simultaneously. The transition to the reductive microenvironments can be related to the activation of SIRT1 and its interaction with NRF2. BCs are utilized to maintain the NAD+/NADH ratio in glucose and lipid metabolism, one of the vital factors for SIRT1 activation. Therefore, BCs are required to guide their precise target functions for optimal actions. More detailed information on the underlying mechanisms in the prevention of dietary stress-induced immune and metabolic disorders needs to be elucidated in future studies.

## Figures and Tables

**Table 1:** BCs regulate cellular redox balance and histone acetylation state.

BCs	Effects	Model	Mechanisms	Ref
Astaxanthin	↓ Oxidative stress ↓ Inflammation	In vitro	↓ ROS, ↓ NOX2 and COX-2	[119,145]
		Macrophage stimulated with LPS and H_2_O_2_	↑ SIRT1, ↑ NRF2, ↑ antioxidant enzymes ↓ NF-κB nuclear translocation	[146,147]
		apolipoprotein E-knockout mice	↑ NRF2, ↑ antioxidant enzymes	[148]
		Hepatic stellate cells	↑ NRF2, ↑ antioxidant enzymes	[149]
		Macrophages stimulated with ethanol	↑ SIRT1, ↑ NRF2, ↑ NAD+ levels	[30]
	↓ hepatic lipid	High-fat diet fed mice	↑ PPARα, ↓ PPARγ and Akt ↓ SREBP1c nuclear translocation	[109,156]
	↑ hepatic autophagy	Cerulein-induced acute pancreatitis in mice	↑ PPARα, ↓ PPARγ and Akt	[151]
Butyrate	↓ Insulin resistance	High-fat diet fed mice	↓ Fasting blood glucose and insulin ↑ PPARα, ↑ AMPK and p38	[160]
	↓ Inflammation	Colorectal cancer	↓ HDACs	[105]
	↑ Hepatic fatty acid oxidation	High-fat diet fed mice HepG2 cells	↓ HDAC3 activity ↑ FGF21 and PGC1α expression	[164]
	↑ Hepatic drug metabolizing ability	HepG2-C3 cells Primary human hepatocytes	↑ Aryl hydrocarbon receptor ↑ Cytochrome p450	[157]
	↑ Thermogenesis in brown fat	High-fat diet fed mice	↑ PGC1α and UCP1,	[156]
	↓ Glucose intolerance Insulin resistance	High-fat diet fed mice	↓ HDAC activity	[160]
	↑ Antioxidant effects in intestine	IEC-6 epithelial cells HT-29 colorectal cancer cells	↑ NRF2, ↓ HDAC activity	[166]
	↓ Inflammation	Human gut lamina propria CD4 T cell Murine splenic cell Human peripheral blood CD4 T cells	↓ HDAC activity ↓ Inflammatory cytokine production ↑ PPARγ signaling pathways	[167,168,169]
Curcumin	↑ Antioxidant	Oxidative or insulin-resistant conditions	↑ Keap1 expression and NRF2	[173]
		Obesity and diabetes	↑ Antioxidant function of NRF2	[174,175]
		Mouse skin Murine epidermal cells	↑ HO-1 expression and NRF2	[177,183]
	↓ Skin tumorigenesis	Mouse skin	↓ COX-2 expression, ↓ NF-κB activity	[176,177]
	↑ Apoptosis	Glioma cell	↓ Histone acetylation, ↓ p300/CBP	[172,178]
	↓ Inflammation	Mantle cell lymphoma High-fat diet-fed rats	↓ Acetylation state ↓ NF-κB activation and inflammation	[179,180]
		High glucose-induced monocyte	↓ NF-κB activity, ↓ IL-6 and TNFα	[181]
	↓ Hyperglycemia and Diabetes	High-fat diet-fed rats	↑ HAT levels, ↑ insulin sensitivity	[180]
		STZ-induced diabetic rats	↓ iNOS and TGF-β1 ↓ p300/CBP and NF-κB	[182]
EGCG	↑ Apoptosis in prostate cancer cells	LNCaP and PC3 cells	↓ HAT activity, ↑ cell cycle arrest ↑ p21/waf1 and Bax	[189]
	↓ Inflammation	High-fat/western-style diet-induced obese mice	↓ HAT activity, ↓ body weight ↓ Hepatic lipids and inflammation	[191]
		High-fat-fed rat (female)	↓ IL-1β and IL-6, ↑ SOD and COMT	[192]
Resveratrol	↓ Inflammation	RAW 264.7 cells JB6 cells	↑ Scavenge free radicals ↓ Lipid peroxidation and DNA damage	[111]
		Paraquat-induced lung injured mice	↑ SIRT1 expression and NRF2 activation	[21]
		Human colon-derived myofibroblast cells	↓ TNFα-induced ROS and ICAM-1 ↑ SIRT1 activation	[196]
		Jurkat lymphoid HeLa and H4 epithelial cells	↓ TNFα-induced NF-κB and MAPK ↓ cytotoxicity and caspase activation	[197]
		Mycobacterium tuberculosis-infected mice	↓ TAK1, NF-κB and MAPK ↑ SIRT1 activation	[200]
		Diabetic rats Mesangial cells	↓ Fibronectin and TGF-β1	[201,202]
NR	↑ Antioxidant ↓ Inflammation	Ethanol-exposed macrophages	↑ SIRT1 activation	[31,205]
	↓ Lipid accumulation	Lieber-DeCarli ethanol liquid diet-fed mice	↑ SIRT1 and PGC1α activation ↑ Mitochondrial function	[48]
	Energy metabolism	Ethanol-exposed macrophages	↑ SIRT1 activation, HIF-1α activation ↓ GLUT1, PDK1, LDHα	[31]
SFN	↑ Antioxidant	Cancer cells	↑NRF2 activation ↑ Detoxifying/antioxidant enzymes	[212]
	↑ Cancer prevention	HCT116 colon cells Human embryonic kidney 293 cells	↓ HDAC2 and HDAC3	[213,215]
		Cancer cell lines	↑ ROS for initiation of apoptosis ↓ ROS during apoptosis ↑ Caspase 3 and 8, ↑ p21 and BAX ↑ NF-κB activation, ↓ HDAC6 activity ↓ HDAC1, 4, 5, and 7 ↑ Histone 3 acetylation, ↑ NRF2	[213,214] [220,223]
	Adipocyte differentiation	3T3-L1 adipocytes	↓ HDAC2, HDAC4, 5, 7, and 9 ↓ PPARγ, FABP4, FAS, ↓ p300 and C/EBPα	[215] [79,218] [225,226]
TGS	↑ Energy metabolism	Cardiomyocytes Skeletal myoblasts Vascular endothelial cells Epithelial cells	↑ Mitochondrial oxygen consumption, ↑ Cellular respiration ↑ ATP production	[233,234]
		Cardiomyocytes and neurons	↑ SIRT1/PGC1α axis, ↑ NRF2 ↑ Mitochondrial biosynthesis	[235,236]
	↓ Inflammation	Inflammatory condition	↓ NF-κB and proinflammatory cytokines	[239,240]
		LPS-stimulated microglia	↓ MAPK phosphorylation, ↑ CREB	241
		LPS-induced RAW 264.7 macrophages	↓ JAK2/STAT3 activation	[230]
		CCl(4)-induced acute liver injured mice	↓ NF-κB subunit p65 and COX-2 ↓ ERK, JNK, and MAPK, p38	[242]
		Thioacetamide-induced liver injured mice D-GalN and LPS-induced liver injured mice	↓ TNFα, IL-1β, IL-6, and caspase-1 ↓ liver fibrosis, ↓ NF-κB, p38/ERK ↑ SIRT1 and NRF2	[243,244]
		D-GalN and LPS-induced acute injured mice	↓ TLR4, ↓ NF-κB and MAPK	[245]
		Cancer cell lines	↓ EGF and EGFR phosphorylation ↓ ERK1/2, p38, and c-JNK activation ↓ ROS/ERK pathway, ↓ ERK-Akt pathway	[249] [250,251] [252,253]
	↑ Anti-diabetes	High-fat diet and STZ-induced diabetic rats	↓ Gluconeogenesis, ↑ glucose transport ↑ insulin production	[254,255]

## Data Availability

Not applicable.
